# Prospective Case-control Study of Contact Tracing Speed for Emergency Department-based Contact Tracers

**DOI:** 10.5811/westjem.2022.5.53196

**Published:** 2022-08-24

**Authors:** Sean C. Weaver, Samuel S. Byrne, Hollianne Bruce, Olivia L. Vargas, Thomas E. Robey

**Affiliations:** * Washington State University Elson S. Floyd College of Medicine, Providence Regional Medical Center Everett, Department of Emergency Medicine, Everett, Washington; † University of Washington Environmental Health and Safety Department, Everett, Washington; ‡ Snohomish Health District, Everett, Washington

## Abstract

**Introduction:**

In Snohomish County, WA, the time from obtaining a positive severe acute respiratory syndrome coronavirus 2 (SARS-CoV-2) test and initiating contact tracing is 4–6 days. We tested whether emergency department (ED)-based contact tracing reduces time to initiation and completion of contact tracing investigations.

**Methods:**

All eligible coronavirus disease 2019 (COVID-19)-positive patients were offered enrollment in this prospective case-control study. Contact tracers were present in the ED from 7 AM to 2 AM for 60 consecutive days. Tracers conducted interviews using the Washington State Department of Health’s extended COVID-19 reporting form, which is also used by the Snohomish Health District (SHD).

**Results:**

Eighty-one eligible SARS-CoV-2 positive patients were identified and 71 (88%) consented for the study. The mean time between positive COVID-19 test result and initiation of contact tracing investigation was 111 minutes with a median of 32 minutes (range: 1–1,203 minutes). The mean time from positive test result and completion of ED-based contact tracing investigation was 244 minutes with a median of 132 minutes (range: 23–1,233 minutes). In 100% of the enrolled cases, contact tracing was completed within 24 hours of a positive COVID-19 test result. For comparison, during this same period, SHD was able to complete contact tracing in 64% of positive cases within 24 hours of notification of a positive test result (P < 0.001). In the ED, each case identified a mean of 2.8 contacts as compared to 1.4 contacts identified by SHD-interviewed cases. There was no statistically significant difference between the percentage of contacts reached through ED contact tracing (82%) when compared to the usual practice (78%) (P = 0.16).

**Conclusion:**

When contact tracing investigations occur at the point of diagnoses, the time to initiation and completion are reduced, there is higher enrollment, and more contacts are identified.

## INTRODUCTION

Rapid testing and contact tracing are foundational for containing rapidly spreading infectious diseases such as coronavirus disease 2019 (COVID-19).^1^,^2^ As with most US health districts, contact tracing in Snohomish County, WA, uses positive test result reports to initiate investigation by the Snohomish Health District (SHD). Typically, the time from testing to investigation completion spans 4–6 days. To reduce this time, we designed a program at the Providence Regional Medical Center Everett (PRMCE) to speed up contact tracing investigations by positioning contact tracers in the emergency department (ED). We hypothesized that physical proximity to the patient and temporal proximity to the diagnosis would decrease the time needed for the contact tracing process. The primary outcome was time to initiation of ED-based contact tracing. Secondary measures included time to investigation completion, number of contacts identified, and percent participation with contact tracing.

## METHODS

This was a prospective case-control study comparing contact tracing times for COVID-19-positive patients in Snohomish County tested in the PRMCE ED to all COVID-19-positive patients in the county, as traced by the SHD standard-of-care process. All patients who tested positive during an eight-week period were offered enrollment. Data collected for the ED cases included timestamps for diagnosis, consent, interview completion, and contact tracing completion. Data collected by the public health department for the standard-of-care group included time of notification and time of completion of contact tracing.

The PRMCE is an urban, tertiary receiving hospital, Level II trauma center, 530-bed community hospital with approximately 86,000 ED visits in 2019^3^ and serves three counties with a total population just over 1,100,000. During the study, the hospital lab used the polymerase chain reaction (PCR)-based rapid GeneXpert platform (Cepheid, Sunnyvale, CA) to diagnose severe acute respiratory syndrome coronavirus 2 (SARS-CoV-2) infection within 90 minutes of nasal swab. Six contact tracers based in the PRMCE ED were trained following SHD recommendations. The contact tracers were a mix of graduate public health students, a second-year medical student with a Master’s in Public Health, and a foreign medical school graduate. A single contact tracer was in the ED between 7 am to 2 am with one hour of overlap from 4 pm to 5 pm to allow for sign-out. From July 10–September 5, 2020 between the hours of 7 am to 2 am, the charge nurse informed the contact tracers of all positive SARS-CoV-2 PCR results. These tests were conducted during routine patient care, independent of the study. Patients with positive results between 2 am to 7 am had their contact information confirmed by nursing staff and were informed that a contact tracer would call them later that day.

Contact tracers were trained under good research practice to ensure research integrity during the process of requesting consent for inclusion in the study. Patients who did not consent to participation were not enrolled and specific information was not collected about them; however, due to the public health emergency, and to minimize biasing the subjects, they were informed they would be contacted later following standard-of-care public health practice. If a patient consented to the study but declined interview due to fatigue or other reason during the ED visit, the contact tracer scheduled a time, preferably within 12 hours, to conduct the interview. Contact tracers conducted interviews using the Washington State Department of Health Extended COVID-19 reporting form to guide the interview.^4^

Tracers were provided caregiver personal protective equipment but minimized contact with patients. Telephones were used to communicate with patients in their rooms, often with tracers standing outside the room’s glass door to further maintain safety. Contacts identified by patients were called immediately. Completed interviews were faxed to the SHD. Confirmed cases from congregate living settings or other complicated situations were faxed to SHD as soon as the interview was complete. Expeditious notification of SHD superseded the goals of the study. The research consent and contact tracing enrollment process took between 5–15 minutes, depending on subject questions. Contact tracers were instructed to obtain follow-up contact information to continue interviews after discharge or admission, so as not to change the patient’s ED length of stay.

**Population Health Research Capsule**

What do we already know about this issue?

*Contact tracing is an important component of public health pandemic response but can be delayed by result reporting and difficult-to-reach populations.*

What was the research question?

*Could proximity to COVID-19 diagnosis decrease the time needed for the contact tracing process?*

What was the major finding of the study?

*Contact tracers for COVID in the ED speed up contact tracing (100% complete within 24 hours vs. 64%), find more contacts (2.8 vs. 1.4) and access populations typically missed by traditional methods.*

How does this improve population health?

*Contact tracing of newly positive patients in the ED can expedite isolation and testing, thereby slowing pathogen spread and reducing population disease burden.*

The primary endpoint of this study was time from result report to initiation of contact tracing. Secondary measurements were time to investigation completion, number of contacts identified, and percent participation with contact tracing. We grouped and compared enrolled subjects’ data against the data provided to the public by the SHD. We calculated 95% confidence intervals (CI) for ED data following the central limit theorem to illustrate numerical distribution of subjects. The SHD provided mean time values and case numbers for percentage calculation. All PRMCE ED patients were included in county-wide SHD values, which biased the SHD data to be more like ED data. We directly compared costs of this study with standard contact tracing costs.

Patients were given the option to enroll in this study (Spokane Institutional Review Board Protocol # STUDY2020000425) or be followed by SHD following public health standard-of-care. Patients not enrolled were included in the publicly reported data provided by SHD. Statistics were calculated using Microsoft Excel (Microsoft Corporation, Redmond, WA) and plotted in Prism (Graphpad Software Inc, La Jolla, CA). We used R v4.0.3 (RStudio Inc, Boston, MA) to conduct a two-sided test at the 95% CI to determine statistical significance.

## RESULTS

**Figure 1:**
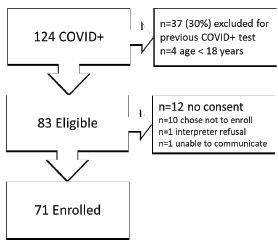
Flowchart of patient enrollment for expedited contact tracing of COVID-19 positive patients beginning in the ED at time of diagnosis. *COVID*, coronavirus disease.

From July 10–September 8, 2020, 124 patients tested positive for SARS-CoV-2. We excluded 37 (30%) patients based on any previous positive COVID-19 test, and four (3%) were excluded due to age (< 18 years old). Of the 83 patients eligible for the study, 10 (12%) declined to enroll in the research protocol and had attempts to contact by SHD based on public health standards of care. One (1%) patient was unable to consent, and one patient (1%) refused to use a hospital-certified interpreter. In total, 71 (86%) eligible patients consented for the study. Figure 1 illustrates enrollment.

The average age of ED patients enrolled in the study was 57 years (95% CI 52–62 years) with a range of 19–94 years. A majority of patients identified their race as White (82%), followed by Asian (6%), unknown (6%), American Indian/Alaskan native (3%), Black/African American (1%), Native Hawaiian or Pacific Islander (1%), and other (1%). This corresponds to the region’s demographics. Primary English-speaking patients accounted for 76% of positive cases followed by Spanish (6%), Ukrainian (6%), nonverbal (6%), Russian (3%), French (1%), Nepali (1%), and Tagalog (1%). Of the enrolled patients, 54% were male, 13% lived in congregate living facilities, including shelters, and 4% identified as homeless on the street. Medicare/Medicaid recipients made up 69% of the enrolled patient population, 21% had private insurance, and 10% were uninsured. Patients presented an average of 4.4 days after symptom onset. In total, 46% enrolled patients were admitted to the hospital and 8% went to the intensive care unit. Follow-up interviews occurred in nine instances (12%), typically at the patient’s request and for admitted patients too weak to communicate.

**Figure 2:**
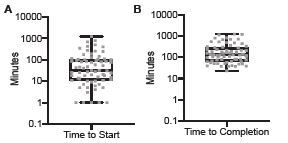
Distributions of times from positive COVID-19 test result when contact tracing begun at time of diagnosis in the emergency department. (A) initiation of contact tracing and (B) investigation completion plotted on a log scale. Box-and-whisker boxes show median and 25th–75th percentiles; whiskers indicate minimum and maximum values. Each individual case is plotted.

The primary outcome under investigation was time to initiation of ED-based contact tracing. The mean time between positive COVID-19 test result and initiation of contact tracing study in the ED was 111 minutes with a median of 32 minutes (range: 1–1,203 minutes). The mean time from positive test result and completion of ED-based contact tracing investigation was 244 minutes with a median of 132 minutes (range: 23–1,233 minutes). Figure 2 illustrates durations of time to contact tracing initiation and investigation completion for each case, including median, quartiles, and range.

When compared to the usual practice, completion of contact tracing within 24 hours was statistically significant. Of the enrolled ED-based contact tracing cases, 100% were completed within 24 hours. During this same time period, the usual practice resulted in 64% of cases being completed in less than 24 hours *(P* = <0.001). In the SHD population, the exact duration between time of positive COVID-19 test result and SHD notification of the positive test result is unknown, but hospital labs and testing sites typically reported results within 6–36 hours. In the ED, each case identified a mean of 2.8 contacts (range 0–9). Cases interviewed by SHD identified an average of 1.4 contacts per case during the same time period. There was not a statistical difference between the percentage of contacts reached through ED-based contact tracing – 83% (162/197), when compared to usual practice, 78% (2,683/3,441) (*P* = 0.16). Of all the contacts ED-based contact tracers were able to reach, only seven (4%) refused to participate.

The ED-based contact tracers were temporary workers paid $25/hour (no benefits) for this two-month study, which is consistent with local cost-of-living salaries. The cost of staffing the ED was $500 per day, which averaged $416 per patient enrolled and $238 per positive case (including cases prospectively excluded). In the two months this study was underway our region saw a small uptick in cases. Just four months after completion of enrollment, the ED saw 8–15 cases per day, which would correspond to $33 to $63 per case, consistent with SHD estimates of $50 per case.

## DISCUSSION

Emergency department-based COVID-19 contact tracing resulted in a decrease in time to initiation and completion of contact tracing. Mathematical modeling has shown that contact tracing will only contribute to containment of COVID-19 if it is conducted with minimal time between symptom onset, positive test result and contact tracing.^5^ A decrease in time to initiation and completion of contact tracing investigation through an ED-based contact tracing program has the potential to have a significant impact on COVID-19 containment. Even with patients declining to enroll in this study, when compared to the usual practice in Snohomish County, a higher participation rate was observed when contact tracing occurred at the point of diagnosis. Endorsement of contact tracing by trusted healthcare professionals, convenience for the patient, and the ability to leverage human interactions are the likely reasons for this benefit.^6^

Multiple factors influence whether a patient participates in contact tracing. Media outlets have documented the struggles health jurisdictions encounter while trying to conduct contract tracing,^7^,^8^ and the practice has been polarizing.^9^ A patient-focused, point-of-diagnosis COVID-19 contact tracing program can mitigate some of these challenges. The trust developed between patient and physician is easily conferred to contact tracers, thereby encouraging participation. Healthcare clinicians also have a clinical understanding of the patient and can identify and address potential barriers to participation. This study indicates that the combination of these factors has the potential to significantly increase patient participation in contact tracing. The research enrollment and contact tracing intervention were brief enough that length of stay was minimally impacted, while the number of contacts identified and the percentage of contacts agreeable to interview was equivalent to the SHD standard of care. As has been reported in other EDs in the United States, we noticed that ED patient volumes decreased throughout most of the pandemic.

Patients typically difficult to reach who are easier to engage in the ED include admitted patients, residents of adult family homes, and people experiencing homelessness. Face-to-face interviews with these individuals, or with their family members, clarified important details in the contact tracing process and resulted in fewer cases lost to follow-up. The cost of contact tracing and its apparent limitations in application to such widespread infections could be mitigated by stationing contact tracers in a safe section of the ED. Contact tracers based in the ED would capture nearly all ED diagnoses and could concurrently work on other contact tracing cases reported through clinics and testing sites. This process would transform the relatively high cost observed in this pilot to a more cost-effective approach. Alternatively, contact tracers could be mobilized to respond to ED-based operations when case numbers dictate effective resource utilization.

## LIMITATIONS

Limitations to this study include the fact that it was a single-center study, the moderate case load, and its cost. An average of 2.1 patients tested positive each day and 1.2 patients were enrolled each day. A higher incidence of cases could overwhelm a single contact tracer resulting in higher loss to follow-up; we estimate that our contact tracers could have reasonably conducted six- to eight-fold the number of cases managed each day during this study’s enrollment. At 1.2 patients per day, the cost per case using a contact tracer salary of $25/hour was $416 per enrolled case. With eight cases per day, the cost per case would be $62.50, which matches the amount paid to traditional contact tracers. The hospital lab turnaround time from swab to result was approximately 90 minutes; this is faster than most facilities in the county, but some patients still found this wait time unacceptable. The SHD received positive test results 24 hours a day; however, overnight results were not processed until morning, with nine fewer hours of coverage than were available to our contact tracers.

At the time of the study, Snohomish County was experiencing a mild surge in cases. Emergency department-based contact tracing could have a greater utility with higher case numbers. The population described in this study matches the local general patient population for race and ethnicity, with sizeable unique underserved populations of Russian- and Ukrainian-speaking individuals, which was the only population over-represented in this study. In Snohomish County 79% of all patients are insured by Medicare or Medicaid, and homelessness is estimated as a factor in 5% of the general ED population. The SHD was unable to contact most of their homeless cases, whereas an ED-based approach enabled contact tracing of these individuals.. While this demographic pertains to the region studied, many elements contributing to challenging follow-up do translate to other catchment hospitals.

## CONCLUSION

This study shows the feasibility of point-of-diagnosis ED-based contact tracing. Implementation required partnership with the administration of Providence Regional Medical Center Everett, the ED nursing staff, and Snohomish Health District leadership. The cost of implementing this project was not overwhelming, and in the context of an outbreak with more COVID-19 cases per day, an economy of scale could reduce the per patient expense. Future efforts should focus on leveraging the power of face-to-face interactions, reducing barriers by capitalizing on technology or a telehealth infrastructure and repurposing healthcare resources to conduct contact tracing at the point of patient contact.

## References

[1] Wu J, Tang B, Bragazzi NL (2020). Quantifying the role of social distancing, personal protection and case detection in mitigating COVID-19 outbreak in Ontario, Canada. J Math Ind.

[2] Centers for Disease Control and Prevention (2020). Coronavirus Disease (COVID-19): Contact Tracing and Investigation Guidelines.

[3] Washington State Hospital Association (2020). Providence Regional Medical Center Everett.

[4] Washington State Department of Health Covid-19 Extended Form, DOH.

[5] Kretzschmar ME, Rozhnova G, Bootsma MCJ (2020). Impact of delays on effectiveness of contact tracing strategies for COVID-19: a modelling study. Lancet Public Health.

[6] National Academies of Sciences, Engineering, and Medicine (2020). Encouraging Participation and Cooperation in Contact Tracing: Lessons from Survey Research.

[7] Khazan O (2020). The most American COVID-19 failure yet. The Atlantic.

[8] Steinhauer J, Goodnough A (2020). Contact tracing is failing in many states Here’s why. The New York Times.

[9] Ollstein AM, Tahir D (2020). Contact tracing foiled by conspiracy theories, lack of federal messaging. Politico.

